# Stereotactic cisternal lavage in patients with aneurysmal subarachnoid hemorrhage with urokinase and nimodipine for the prevention of secondary brain injury (SPLASH): study protocol for a randomized controlled trial

**DOI:** 10.1186/s13063-021-05208-6

**Published:** 2021-04-15

**Authors:** Roland Roelz, Fabian Schubach, Volker A. Coenen, Carolin Jenkner, Christian Scheiwe, Jürgen Grauvogel, Wolf-Dirk Niesen, Horst Urbach, Christian Taschner, Jochen Seufert, Jürgen Kätzler, Jürgen Beck, Peter C. Reinacher

**Affiliations:** 1 Department of Neurosurgery, Medical Center – University of Freiburg, Faculty of Medicine, University of Freiburg, Freiburg im Breisgau, Germany; 2 Clinical Trials Unit, Medical Center – University of Freiburg, Faculty of Medicine, University of Freiburg, Freiburg im Breisgau, Germany; 3 Department of Stereotactic and Functional Neurosurgery, Medical Center – University of Freiburg, Faculty of Medicine, University of Freiburg, Freiburg im Breisgau, Germany; 4 Department of Neurology and Neurophysiology, Medical Center – University of Freiburg, Faculty of Medicine, University of Freiburg, Freiburg im Breisgau, Germany; 5 Department of Neuroradiology, Medical Center – University of Freiburg, Faculty of Medicine, University of Freiburg, Freiburg im Breisgau, Germany; 6 Department of Medicine II, Division of Endocrinology and Diabetology, Medical Center – University of Freiburg, Faculty of Medicine, University of Freiburg, Freiburg im Breisgau, Germany; 7 Department of Internal Medicine III, Clinical Trials Office, University Medical Center Ulm, Ulm, Germany; 8 Fraunhofer Institute for Laser Technology (ILT), Aachen, Germany

**Keywords:** Delayed cerebral infarction (DCI), Aneurysmal subarachnoid hemorrhage (aSAH), Clinical trial, Stereotactic ventriculocisternostomy (STX-VCS), Urokinase, Nimodipine, Intracisternal lavage, Intrathecal treatment

## Abstract

**Background:**

Delayed cerebral infarction (DCI) is a major cause of death and poor neurological outcome in patients with aneurysmal subarachnoid hemorrhage (aSAH). Direct intrathecal therapies with fibrinolytic and spasmolytic drugs have appeared promising in clinical trials. However, access to the subarachnoid space for intrathecal drug administration is an unsolved problem so far, especially in patients with endovascular aneurysm securing. We investigate a therapy protocol based on stereotactic catheter ventriculocisternostomy (STX-VCS), a new approach to overcome this problem. The primary objective of this study is to assess whether cisternal lavage with urokinase, nimodipine, and Ringer’s solution administered via a stereotactically implanted catheter into the basal cisterns (= investigational treatment (IT)) is safe and improves neurological outcome in patients with aSAH.

**Methods:**

This is a randomized, controlled, parallel-group, open-label phase II trial. Fifty-four patients with severe aSAH (WFNS grade ≥ 3) will be enrolled at one academic tertiary care center in Southern Germany. Patients will be randomized at a ratio of 1:1 to receive either standard of care only or standard of care plus the IT. The primary endpoint is the proportion of subjects with a favorable outcome on the Modified Rankin Scale (defined as mRS 0–3) at 6 months after aSAH. Further clinical and surrogate outcome parameters are defined as secondary endpoints.

**Discussion:**

New approaches for the prevention and therapy of secondary brain injury in patients with aSAH are urgently needed. We propose this RCT to assess the clinical safety and efficacy of a novel therapy protocol for intrathecal administration of urokinase, nimodipine, and Ringer’s solution.

**Trial registration:**

Deutsches Register Klinischer Studien (German Clinical Trials Register), DRKS00015645. Registered on 8 May 2019

## Administrative information

The order of the items has been modified to group similar items (see http://www.equator-network.org/reporting-guidelines/spirit-2013-statement-defining-standard-protocol-items-for-clinical-trials/).

**Table:** 

**Title {1}**	Stereotactic Cisternal Lavage in Patients with Aneurysmal Subarachnoid Hemorrhage with Urokinase and Nimodipine for the Prevention of Secondary Brain Injury (SPLASH): study protocol for a randomized controlled trial
**Trial registration {2a and 2b}**	EudraCT: 2017-000868-15 Deutsches Register Klinischer Studien (German Clinical Trials Register), DRKS00015645, registered 8 May 2019, https://www.drks.de/drks_web/navigate.do?navigationId=trial.HTML&TRIAL_ID=DRKS00015645 All items from the WHO Trial Registration Dataset can be found both within the DRKS entry and within this manuscript.
**Protocol version {3}**	Version 2.3, dated 16 Aug 2019
**Funding {4}**	The study is funded by a research grant from Else Kröner-Fresenius-Stiftung (EKFS), a German charitable foundation (grant recipients: Dr. Roelz, Dr. Reinacher; award no.: 2016_A208). Dr. Roelz is funded by the Berta-Ottenstein-Programme for Clinician Scientists, Faculty of Medicine, University of Freiburg.
**Author details {5a}**	Roland Roelz^1#^, Fabian Schubach^2#^*, Volker A. Coenen^3^, Carolin Jenkner^2^, Christian Scheiwe^1^, Jürgen Grauvogel^1^, Wolf-Dirk Niesen^4^, Horst Urbach^5^, Christian Taschner^5^, Jochen Seufert^6^, Jürgen Kätzler^7^, Jürgen Beck^1^, Peter C. Reinacher^3,8^ ^1^ Department of Neurosurgery, Medical Center – University of Freiburg, Faculty of Medicine, University of Freiburg, Germany ^2^ Clinical Trials Unit, Medical Center – University of Freiburg, Faculty of Medicine, University of Freiburg, Germany ^3^ Department of Stereotactic and Functional Neurosurgery, Medical Center – University of Freiburg, Faculty of Medicine, University of Freiburg, Germany ^4^ Department of Neurology and Neurophysiology, Medical Center – University of Freiburg, Faculty of Medicine, University of Freiburg, Germany ^5^ Department of Neuroradiology, Medical Center – University of Freiburg, Faculty of Medicine, University of Freiburg, Germany ^6^ Department of Medicine II, Division of Endocrinology and Diabetology, Medical Center – University of Freiburg, Faculty of Medicine, University of Freiburg, Germany ^7^ University Medical Center Ulm, Department of Internal Medicine III, Clinical Trials Office, Ulm, Germany ^8^ Fraunhofer Institute for Laser Technology (ILT), Aachen, Germany * Correspondence: fabian.schubach@uniklinik-freiburg.de ^#^ These authors contributed equally to this work.
**Name and contact information for the trial sponsor {5b}**	Medical Center – University of Freiburg, represented by the Chief Medical Officer (CMO) (Leitender Ärztlicher Direktor), Breisacher Str. 153, 79110 Freiburg, Germany
**Role of sponsor {5c}**	The funder of the study (EKFS) has no role in designing the study protocol or in acquiring, analyzing, interpreting or publishing study data. The study sponsor (Medical Center – University of Freiburg) and its delegates, most notably, the principal investigator and his deputy, have full control over the collection, management, analysis, and interpretation of data, the writing of the report, and the decision to submit the report for publication.

## Introduction

### Background and rationale {6a}

Aneurysmal subarachnoid hemorrhage (aSAH) results from the rupture of an intracranial aneurysm. It represents a medical catastrophe as it confers a high rate of poor neurological outcome and death [1].

Secondary brain injury contributes significantly to poor outcome [2, 3]. In contrast to brain injury incurred during the initial bleeding event, secondary brain injury is potentially amenable to medical treatments. The major mechanism of secondary brain injury is delayed cerebral infarction (DCI) [4, 5]. Days after aSAH, blood break-down products induce prolonged constriction of the cerebral arteries (cerebral vasospasm, CVS) leading to cerebral hypoperfusion and DCI [6, 7]. Owing to the importance of CVS and DCI for the prognosis of aSAH patients, management of CVS is the central treatment focus after aneurysm securing.

Among current treatment options to prevent CVS, the only one to reach level I evidence is oral administration of nimodipine [8, 9]. However, the efficacy is limited [8]. Treatment strategies in established CVS, such as hemodynamic augmentation and endovascular interventions, are commonly applied in clinical practice but are not supported by reliable evidence and come with considerable medical risks (e.g., arterial perforations/dissections, hemorrhagic transformation of brain infarcts, brain edema, cardiovascular events) [10, 11]. Beyond CVS, hydrocephalus is a common sequela of aSAH, typically caused by subarachnoid, intraventricular, and intraparenchymal blood obstructing the cerebrospinal fluid (CSF) pathways, with 6–45% of patients depending on permanent CSF diversion by shunting as a result [12]. Shunt-dependent hydrocephalus is clearly associated with inferior functional outcomes [12, 13]. In summary, despite all efforts, prevention and therapy of secondary brain injury are unsolved medical problems as current treatment options are frequently ineffective.

Intrathecal therapy has been investigated as an alternative treatment approach to interrupt the mechanisms of secondary brain injury for over 30 years [14]. The rationale for intrathecal administration of fibrinolytic drugs, such as urokinase or recombinant tissue plasminogen activator (rtPA), is to remove subarachnoid blood clots and thus the trigger for CVS, while spasmolytic drugs such as nimodipine target the vessels affected by vasospasm directly. Compared to systemically administered drugs, intrathecal delivery brings these agents immediately into contact with their respective target of action. Several clinical trials (mostly non-randomized) have investigated this approach and suggest the general feasibility, safety, and—in part—considerable efficacy of direct intrathecal drug delivery [14].

However, these trials face one major shortcoming: treatments were almost exclusively performed in patients with clipping of the ruptured aneurysm which provides surgical access to the subarachnoid space for implementation of intrathecal therapy. Yet, the majority (approx. 70%) of ruptured aneurysms today is secured by endovascular techniques where no direct access to the brain (e.g., for the placement of a cisternal catheter) is present. Access routes explored so far have proven as either ineffective (e.g., intraventricular drug administration via an external ventricular drain (EVD) [15]) or were associated with significant risks (e.g., drug administration via lumbar catheters [16]). Hence, alternative access routes to the basal cisterns of the brain, the anatomical target region for intrathecal treatments, are required.

To address this “access problem,” we introduced a stereotactic neurosurgical method for catheter implantation into the basal cisterns, stereotactic catheter ventriculocisternostomy (STX-VCS). Based on this method of access, we developed a sequential therapy protocol for cisternal lavage and intrathecal drug delivery. This approach was first applied between September 2015 and October 2016 in individual treatments in 20 patients with aSAH in our institution, with promising results in a retrospective analysis [17]. The access method itself, timing of the intervention, patient selection, sequence of application, and optimal dosing of drugs were refined to prepare the present RCT.

### Objectives {7}

The primary objective of this trial is to assess whether sequential cisternal lavage with urokinase, nimodipine, and Ringer’s solution administered via a stereotactically implanted catheter into the basal cisterns (investigational treatment (IT)) improves neurological outcome in patients with aSAH compared to standard treatment, as measured by the Modified Rankin Scale (mRS).

Key secondary objectives include the assessment of the effect of the IT on rates of DCI and related complications, its effect on further clinical and surrogate endpoints, and its safety. Table 1 presents the secondary objectives and endpoints in detail.

**Table 1:** Secondary objectives and endpoints

Secondary objectives	Secondary endpoints
To assess the effect of the IT on neuropsychological outcome	**Neuropsychological outcome** at 6 months following aSAH: - Cognitive performance (Montreal Cognitive Assessment) - Health-related quality of life (SF-36) - Fatigue, anxiety, and depressive symptoms (Frontal Systems Behavior Scale, Multidimensional Assessment of Fatigue, Hospital Anxiety and Depression Scale) - Post-traumatic stress disorder (Impact of Event Scale–R) Return-to-work parameters at 6 months
To assess the effect of the IT on DCI	Rate and severity of **DCI** according to the Vergouwen criteria
To assess the effect of the IT on **delayed ischemic neurological deficits (DIND)** after aSAH	Rate of delayed ischemic neurological deficit (DIND), defined as **clinical deterioration caused by delayed cerebral ischemia (i.e., a new focal neurological deficit or decline on the Glasgow Coma Scale of 1 point not attributable to other causes)** on days 3–21
To assess the effect of the IT on the development of post-hemorrhagic hydrocephalus	Rates of shunt-dependent **hydrocephalus** at 6 months following aSAH
To assess the effect of the IT on sonographic vasospasm	**Delta mean flow velocities** of both middle cerebral arteries—measured by transcranial Doppler ultrasonography on days 3–21
To assess the effect of the IT on the need for endovascular interventions for the treatment of cerebral vasospasm	Rate of **endovascular interventions** for the treatment of cerebral vasospasm
To assess the effect of the IT on the course of intensive care therapy	**Key parameters of intensive care medicine (Sequential Organ Failure Score)**
To assess the effect of the IT on **morphological brain damage**	**Morphological brain damage** at 6 months after aSAH as assessed by MRI
To assess the effect of the IT on further clinical and surrogate outcomes	NIHSS score at days 3–21 and at 6 months
	Key parameters of **endocrinological dysfunction**
	**Key markers of neuronal injury and systemic inflammation in patient blood**
	**Electroencephalographic patterns as measured by continuous EEG monitoring during intensive care period (exploratory endpoint)**
To assess the **safety of the IT**	**Safety of IT:** (serious) adverse events related to the IT

### Trial design {8}

**Fig. 1:**
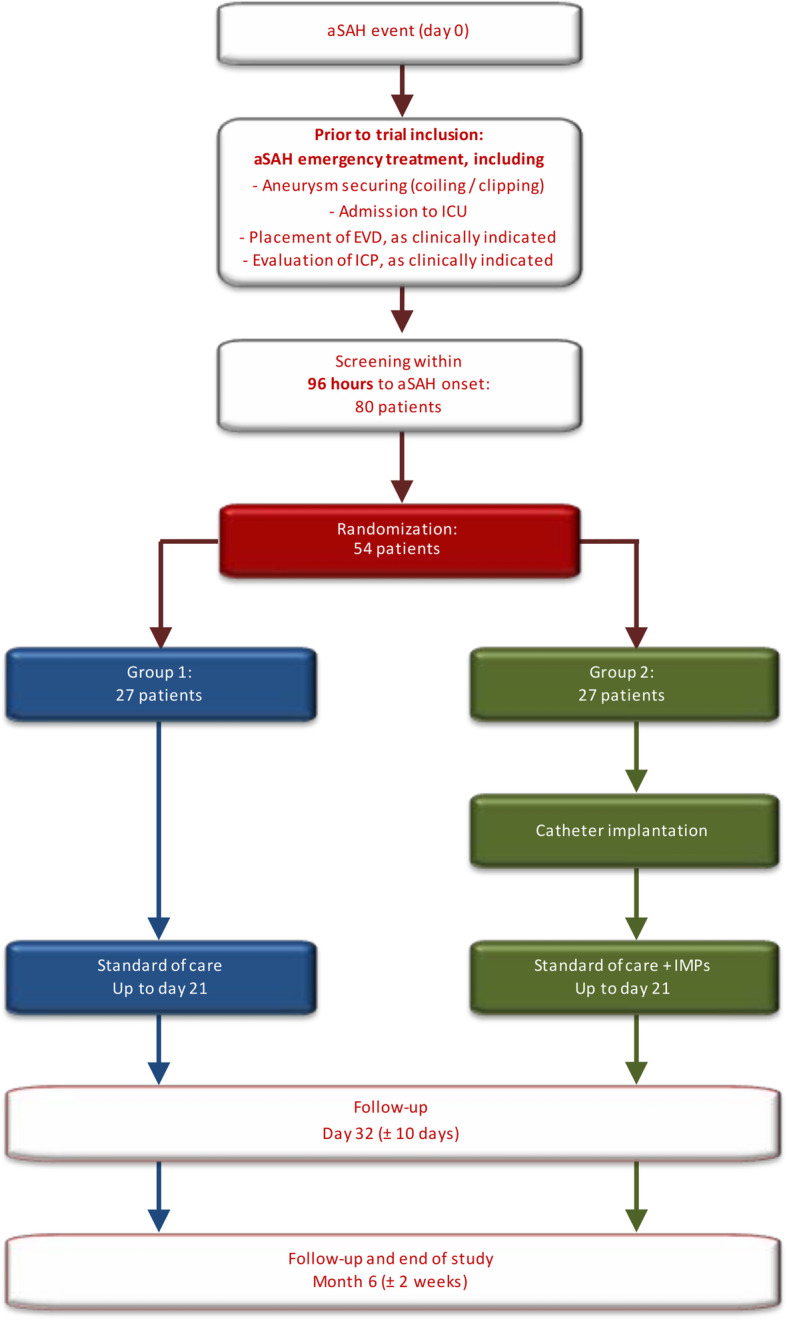
Study design

This is a mono-center, randomized, controlled, parallel-group, open-label phase II trial. Fifty-four patients will be randomized at a ratio of 1:1 to receive either standard of care or standard of care plus the IT (Fig. 1).

## Methods: participants, interventions, and outcomes

### Study setting {9}

Patients with severe aSAH (WFNS ≥3) will be enrolled at the Medical Center, University of Freiburg, a large academic tertiary care center in Southern Germany. Patients will be recruited from the population of patients referred to our institution.

Both groups will receive the required emergency treatment, most notably securing of ruptured brain aneurysm(s) by microsurgical clipping or endovascular coiling, as soon as possible after admission to hospital, prior to randomization, i.e., independent of trial participation. Likewise, the decision for placement of an external ventricular drain (EVD) will be made as medically indicated, i.e., as part of routine treatment outside of the trial. The same is true for the evaluation of intracranial pressure (ICP). In our institution, all patients with severe aSAH usually receive invasive ICP monitoring.

### Eligibility criteria {10}

Patients eligible for inclusion in this trial must meet all the inclusion criteria and none of the exclusion criteria listed in Table 2.

**Table 2:** In-/exclusion criteria

**Inclusion criteria**	1. Male or female patients aged ≥18 years and < 80 years 2. Modified Fisher grade 3 or 4 3. Cisternal/ventricular blood amount according to Hijdra score ≥ 20 4. Admission WFNS grade ≥ 3 (if grade 5 only with fixed dilated pupil due to raised ICP for less than 45 min) 5. External ventricular drain (EVD) in situ or indication for placement of EVD 6. Disease duration ≤96 h before randomization 7. Written informed consent, either by the patient or by the patient’s legally authorized representative 8. Cerebral aneurysm as a definitive source of subarachnoid hemorrhage 9. Patients in whom the cerebral aneurysm has been safely treated via open surgical or endovascular technique
**Exclusion criteria**	1. Pregnancy 2. Surgical contraindications according to the opinion of the investigator 3. Inability to administer study medication (known allergy to urokinase or nimodipine) 4. Presence of a severe illness prior to aSAH (e.g., progressive cancer, terminal organ failure, severe neurological disorder, life expectancy < 1 year) 5. Known and persistent abuse of medication or drugs 6. Presence of severe cerebral infarction related to the aSAH or medical procedures prior to randomization 7. Presence of intracerebral hematoma that is ≥30 ml (assessed using the AxBxC/2 method) or in eloquent location prior to randomization 8. Presence of a condition or abnormality that in the opinion of the investigator would compromise the safety of the patient 9. Known severe complications during aneurysm securing (e.g., dissections of blood vessels, vessel occlusions, re-hemorrhage) 10. Clinical signs of brain stem/midbrain compression (dilated pupil not reacting to light) persisting for more than 45 min at any time between aSAH onset and randomization 11. Persons who are in a relationship of dependence/employment with the sponsor or the investigator 12. For MRI follow-up: cardiac pacemaker and/or cardiac defibrillator. Stent implantation within the last 6 weeks prior to MRI, claustrophobia

### Who will take informed consent? {26a}

Written informed consent will be obtained for every subject participating in this clinical trial. However, most of the patients will not be able to give informed consent initially due to the severity of the underlying aSAH. Therefore, three different informed consent procedures will be distinguished in this trial:

- Patient’s own informed consent
- Informed consent by the patient’s legal representative
- Patient’s presumed consent

The patient’s own informed consent will be obtained whenever possible. If this is not possible, consent by the patient’s legal representative will be obtained. If neither of the two is possible, the patient may be included based on his/her presumed consent.

An authorized investigator will be responsible for explaining the nature, significance, implications, expected benefits, and potential risks of the clinical trial and alternative treatments to the patient or his/her representative and for obtaining written informed consent.

To include a patient into the study based on presumed consent, the following preconditions must be met:

- The patient him-/herself is unable to provide informed consent.
- A legal representative is not in place and cannot be established in adequate time.
- An emergency situation as per the German Medicines Act (section 41) is given.

As additional precautionary measures to be taken in such a case, the patient’s presumed will must be determined, e.g., by consulting the patient’s next of kin. Furthermore, an independent physician who is not involved in the trial must be present during the discussion for determining the patient’s presumed will.

If a patient’s trial inclusion has initially been based on consent by a legal representative or on presumed consent, the patient’s own written informed consent will be obtained as soon as the patient’s condition allows for an informed decision.

### Additional consent provisions for collection and use of participant data and biological specimens {26b}

Patients or their legal representatives will be given the opportunity to voluntarily provide additional consent (opt-in) for having blood samples stored in a local biobank (see item {33} for details).

## Interventions

### Explanation for the choice of comparators {6b}

The safety and efficacy of the IT are to be investigated as an add-on to the best available medical treatment as per applicable treatment guidelines and institutional routine.

### Intervention description {11a}

Patients randomized to group 1 will receive standard-of-care treatment for patients with aSAH according to the European guidelines [18]. Continuous ICP monitoring will be performed throughout aSAH treatment in order to detect pathological increases of ICP.

**Fig. 2:**
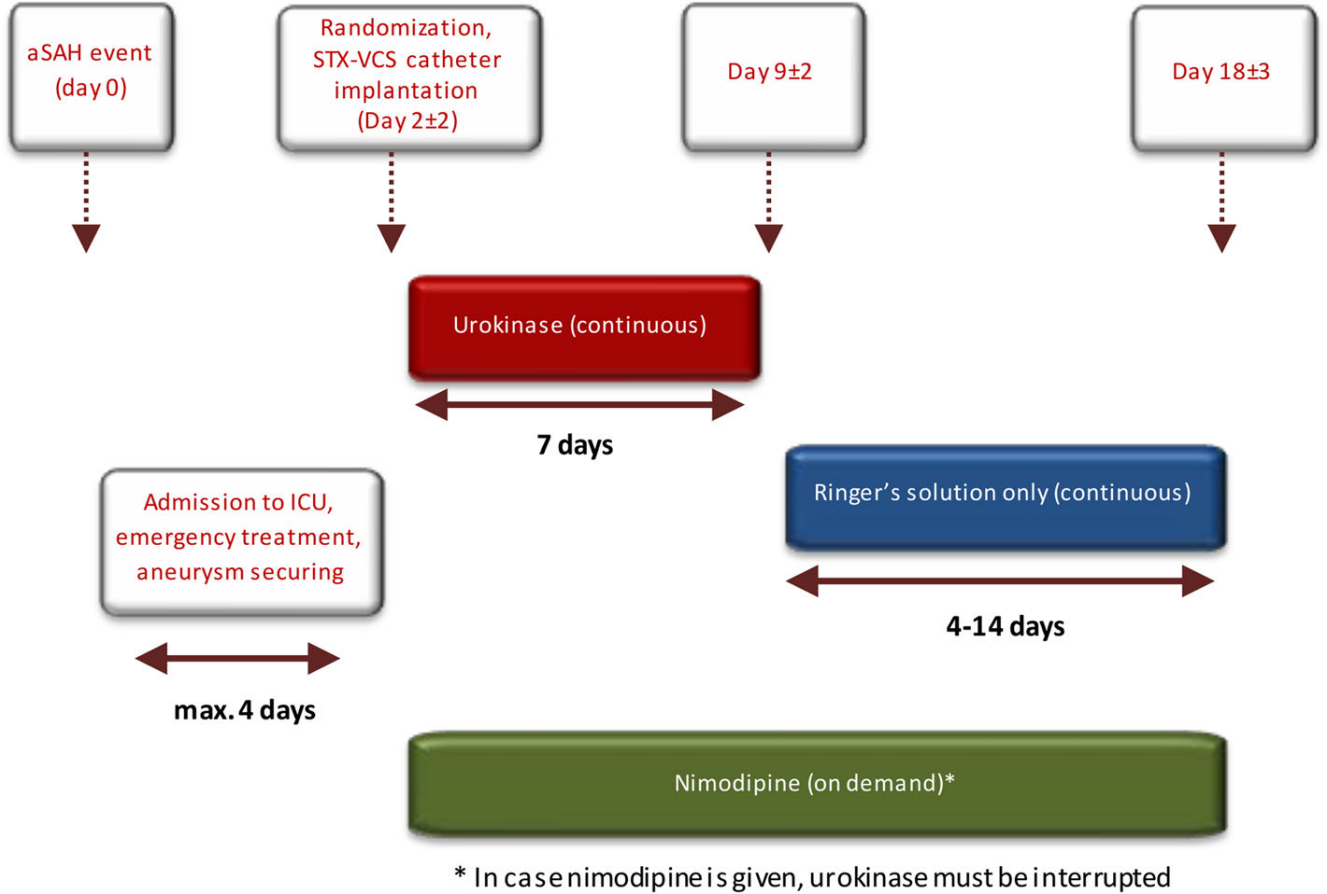
Therapy protocol in group 2

Patients randomized to group 2 will receive standard-of-care treatment plus additional IT. Essentially, the IT consists of a sequential cisternal lavage with urokinase for 7 days starting from randomization, then with Ringer’s solution for another 4 to 14 days, and with nimodipine anytime between randomization and day 21 in case of verified or suspected CVS, administered via a stereotactically implanted catheter. Ringer’s solution will be used as a carrier solution for the other two investigational medicinal products (IMPs), urokinase and nimodipine, and as a third IMP. A graphic overview of the therapy protocol in group 2 is provided in Fig. 2 and a detailed dosing regimen in Table 3.

**Table 3:** Dosing regimen of investigational medicinal products

Study medication	Pharmaceutical form and route of administration	Duration/regimen
Urokinase diluted in Ringer’s solution	Intrathecal use	Continuous cisternal lavage with 100 I.U. urokinase per ml Ringer’s solution at a rate of ~ 50 ml/h for 7 days following implantation of the catheter, i.e., from day 2 ± 2 until day 9 ± 2 after aSAH, or until the onset of cerebral vasospasm or DIND. Total daily dose: 120.000 I.U. Note: urokinase administration has to be stopped when nimodipine is given.
Nimodipine diluted in Ringer’s solution	Intrathecal use	Nimodipine is given on demand in case of vasospasm (i.e., delayed ischemic neurological deficit or mean flow velocities of any intracranial vessel > 120 cm/s on Doppler ultrasonography). In this case, nimodipine is administered as continuous cisternal lavage with 0.005 mg nimodipine per ml Ringer’s solution at a rate of ~ 50 ml/h. Nimodipine infusion is performed from the onset of vasospasm to the cessation of vasospasm, at least, however, for 24 h. If necessary, nimodipine can be administered as often as necessary or for extended periods during aSAH treatment (up to the entire aSAH treatment phase of this trial, i.e., up to 21 days). Total daily dose: 6 mg. Note: urokinase administration has to be stopped when nimodipine is given.
Ringer’s solution	Intrathecal use	Continuous cisternal lavage with Ringer’s solution at a rate of ~ 50 ml/h, starting from day 9 ± 2 (i.e., from the end of urokinase therapy) to day 18 ± 3 after aSAH; the exact stop date will be determined based on the investigator’s risk-benefit assessment for the individual patient. The criteria for this assessment are risk of catheter infection, macroscopic clearance of lavage fluid (i.e., no or minimal residual xanthochromia), and individual risk assessment for the development of vasospasm beyond day 15, considering patient neurological status, WFNS grade, sex, age, blood amount, and course of transcranial Doppler ultrasonographic measurements.

The application of the IT requires a stereotactic neurosurgical intervention (STX-VCS) for catheter placement after aneurysm securing and randomization (on day 2 ± 2, usually day 2 or 3, ≤96 h after aSAH). Pre- and postoperative imaging (cCT) will be performed for the planning of surgery, verification of appropriate catheter position, and ruling out postoperative complications.

**Fig. 3:**
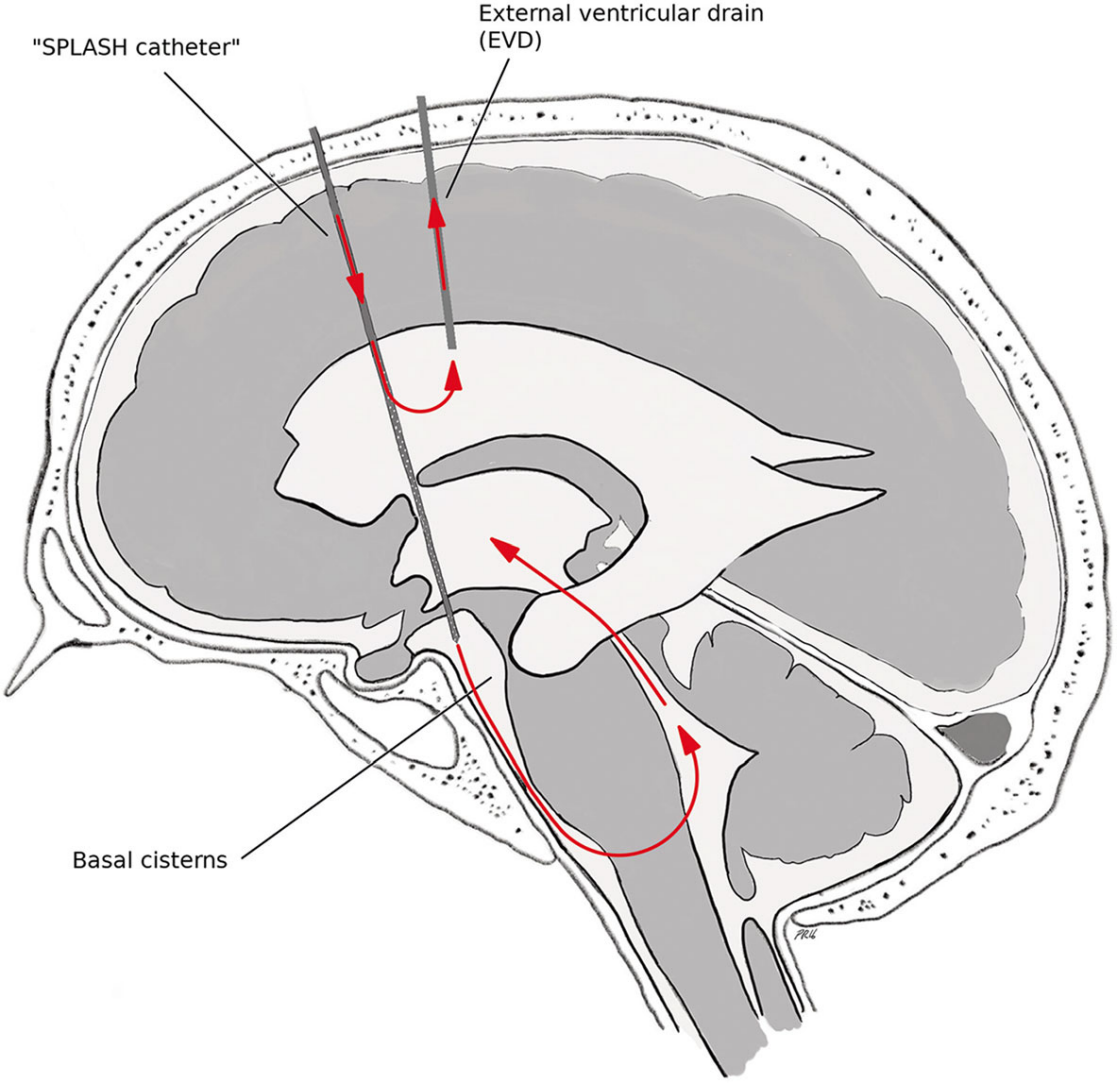
Setup of investigational treatment administration. Arrows indicate the flow direction of the lavage solution

The STX-VCS procedure has been described in detail elsewhere [17, 19]. In the present clinical trial, the STX-VCS catheter is used for infusion of the IMPs, thus representing the inflow tract. A separate EVD—given to every patient with severe aSAH as a matter of clinical routine—represents the outflow tract. Figure 3 shows a schematic drawing of the setup during the administration of the IT.

### Criteria for discontinuing or modifying allocated interventions {11b}

Investigators must follow the protocol for administering the IT whenever possible. However, modifications may be permitted when the patients’ safety or well-being is concerned. Table 4 lists the details on the modifications that will be permitted and under which circumstances.

**Table 4:** Modification of investigational treatment

Event/problem	Permitted modification
Procedure-related undesired effect, i.e., attributable to physical/mechanical effects of intrathecal fluid application	Reduction of infusion rate while maintaining the doses of the IMPs.
Undesired effect that is potentially attributable to pharmacological effects of urokinase or nimodipine	Reduction in doses of these drugs or lavage with Ringer’s solution only.
Occurrence of intracranial hemorrhage after stereotactic catheter implantation and administration of IMP	• Evaluation of whether hemorrhage is related to IMP administration, related to the application procedure, or due to other causes (e.g., due to aneurysm re-bleed, secondary aneurysm rupture, surgical procedures, hemorrhagic transformation of cerebral infarction, spontaneous). • Termination of urokinase and nimodipine if administration of these IMPs is the most likely cause of intracranial hemorrhage. It will not be recommenced until deemed safe by the investigator. • Lavage with Ringer’s solution only if deemed safe by the investigator. • If relatedness to IMPs considered unlikely by the investigator: evaluation of potential benefits and risks of continuation of the IMPs; continued administration of IMPs if benefits of continuation are assumed to outweigh the risks.
Occurrence of raised intracranial pressure (ICP > 20 mmHg)	If considered related to IT administration: termination until normalization of ICP; continued administration of IMPs if deemed safe by the investigator.
Occurrence of cardiac symptoms (e.g., brady- or tachycardia, arrhythmias, electrocardiographic abnormities)	• If graded as serious and if potentially induced by the IT, administration of the IMPs is paused immediately and may be continued with or without dose modifications and changes in infusion rates. • If graded as non-serious but related to the IT: continuation of IT with dose modifications and/or changes in infusion rates.
Occurrence of headache	Reduction of IMP doses and/or infusion rates if considered attributable to the IT.
Normal route of lavage (intracranial infusion via the cisternal STX-VCS catheter and outflow via the EVD) not possible, e.g., due to impaired outflow	Reversed flow direction (i.e., EVD in, cisternal catheter out).
Lavage therapy not feasible at the intended infusion rate	Lower infusion rate or small volume bolus lavage therapy.
Particular clinical circumstances (e.g., recurrent intracranial hemorrhage, persisting intraventricular/intracerebral/subarachnoid blood clots, refractory cerebral vasospasm, cerebral vasospasm lasting beyond day 21)	Recommencement and/or dose modifications of intrathecally administered urokinase or nimodipine.

### Strategies to improve adherence to interventions {11c}

As this is a trial involving intensive care patients, adherence to the intervention protocols in the treatment phase lies primarily with the investigators.

### Relevant concomitant care permitted or prohibited during the trial {11d}

Concomitant anticoagulant therapy must be discontinued for the stereotactic procedure. Otherwise, there are no restrictions regarding concomitant treatment.

### Provisions for post-trial care {30}

Treatment and imaging follow-up after the end of the trial (e.g., for long-term aneurysm surveillance) will be performed according to the trial site’s routine. Otherwise, post-trial treatment is at the discretion of the treating physician. As given by the German Medicines Regulation, an insurance cover exists for all trial participants for compensation to those who suffer harm from trial participation.

### Outcomes {12}

The primary endpoint is the proportion of subjects with a favorable outcome as measured on the Modified Rankin Scale (mRS) at 6 months after aSAH, assessed by an independent physician. mRS will be analyzed in a dichotomized fashion:

- Favorable, defined as mRS 0–3 (independent)
- Unfavorable, defined as mRS 4–6 (dependent/dead)

Secondary endpoints are provided in Table 1.

### Participant timeline {13}

A comprehensive visit flowchart detailing all study-related assessments and their schedule is presented in Additional File 1.

### Sample size {14}

The sample size calculation is based on the primary endpoint dichotomized mRS (mRS > 3) with an expected proportion of 50% in the standard-of-care group and 15% in the experimental treatment group. These assumptions are based on the first results of individual off-label treatments with the new procedure as described by Roelz et al. [17, 19].

The study is planned to detect a difference between the experimental and control groups with respect to dichotomized mRS (mRS > 3) with a power of 80% using the two-sided *χ*^2^ test at a two-sided significance level of 5%. The null hypothesis is rejected if the asymptotic two-sided 95% confidence interval for the odds ratio (control vs. experimental) does not contain one. Under these assumptions, 54 patients are required for analysis.

### Recruitment {15}

All patients with aSAH at the study site will be screened for inclusion into the study. If a patient appears to be eligible for the trial, written informed consent by the patient or by his or her legal representative will be sought. Otherwise, no specific recruitment strategies are possible in this trial involving emergency patients.

## Assignment of interventions: allocation

### Sequence generation {16a}

Randomization will be performed at a ratio of 1:1. The randomization code will be generated using the electronic, web-based tool randomizer.at (https://randomizer.at/). Block randomization will be performed. The block length will be documented separately and will not be disclosed to the site.

### Concealment mechanism {16b}

Randomization using the above mentioned service is performed immediately before carrying out the trial intervention and only after all screening assessments have been performed. The service will not release any randomization information before this time, thus ensuring adequate allocation concealment.

### Implementation {16c}

An authorized investigator will randomize any given trial patient by logging on to randomizer.at immediately before carrying out the trial intervention (control vs. experimental treatment). The treatment arm assigned to the patient will be displayed instantly on the screen and sent by e-mail in addition.

## Assignment of interventions: blinding

### Who will be blinded {17a}

Not applicable, as this is an open-label trial.

### Procedure for unblinding if needed {17b}

Not applicable, as this is an open-label trial.

## Data collection and management

### Plans for assessment and collection of outcomes {18a}

A description and explanation of study instruments can be found within the comprehensive flowchart in Additional File 1.

### Plans to promote participant retention and complete follow-up {18b}

No specific plans to promote participant retention are implemented in this trial. As this is a trial involving intensive care patients, participant retention is usually not an issue in the treatment phase. In surviving patients, the experience with achieving follow-up for at least 6 months is also very good.

### Data management {19}

A professional and validated data management system that is compliant with Good Clinical Practice (GCP) will be used. Details on the data management system and on measures to promote data quality are provided in Table 5.

**Table 5:** Data management system

**General and technical features**	• Name: RDE-LIGHT version 1.5 • Compliance with good clinical practice (ICH E6 GCP) • Presence of an audit trail • Proprietary remote data entry system based on HTML forms • Developed, validated and maintained by the Clinical Trials Unit, Medical Center, University of Freiburg
**Additional documents to be created for more detailed documentation**	• Data management plan for details on procedures, responsibilities, etc. • Database plan for the description of technical specifications of the database and the e-forms (variable names, attributes, and data entry checks) • Data validation plan • Data management report for documentation of performance of data management and deviations from the data management plan, if any
**Additional measures to promote data quality and integrity**	• Programming of range checks for entered values at the CRF level • Validation of the trial database and edit checks of the e-forms before any data entry is performed • Documented training of any data entry personnel before access to the trial database is given • Review of the data for completeness, consistency, and plausibility using the SAS software. The checks to be programmed will be specified beforehand in the data validation plan. • Implementation of a query management, i.e., after running the check programs, the resulting queries will be sent to the investigator for correction or verification of the documented data • Validation of all programs that can be used to influence the data or data quality (e.g., data validation programs, programs for CRF/query tracking, programs for import of RDE-LIGHT data into SAS or for import of external data) • Source data verification (SDV) checks by the clinical research associate (100% SDV checks for critical data points)

### Confidentiality {27}

Information about trial patients will be kept confidential and will be managed under the applicable laws and data protection legislation. The data collected during the study will be collected, stored, and evaluated in pseudonymized form only, i.e., every trial participant is assigned a study-specific pseudonymous identification code. Only authorized trial personnel at the trial site have access to confidential information by which the identity of a patient can be revealed. The data collection system uses built-in security features to prevent unauthorized access to confidential participant information.

### Plans for collection, laboratory evaluation, and storage of biological specimens for genetic or molecular analysis in this trial/future use {33}

Blood samples will be retained and stored in a secure place at the trial site for future analyses of potential systemic effects of aSAH therapy. Specifically for this purpose, a local biobank will be established at the trial site. To this end, three blood samples per patient (approx. 20 ml each) will be collected along with the other trial-related blood drawings on day 2 ± 2, day 7, and day 14. The samples will be stored at − 80 °C and will be destroyed after 10 years. Institutional guidelines for the maintenance, use, and destruction of samples will be adhered to. In case specific analyses are to be performed from these samples, this will be done in separate research projects for which a separate approval from the responsible ethics committee will be requested.

## Statistical methods

### Statistical methods for primary and secondary outcomes {20a}

The effects of the experimental vs. control intervention with respect to the primary endpoint will be estimated in the intention-to-treat (ITT) population by logistic regression. As an estimate of the effect size, the odds ratio will be given with a corresponding asymptotic two-sided 95% confidence interval. The two-sided test on the difference between the two trial arms at significance level 5% will be based on the corresponding asymptotic two-sided 95% confidence interval from the logistic regression model. Data should be collected regardless of the patient’s adherence to the protocol, especially on the clinical outcome, to obtain the best approximation to the full analysis set. Data should also be collected on other therapies received post-dropout. Specifically, full details of the type of additional (non-randomized) therapy given, including when and for how long it was used and at what dose, should be collected. In case of missing values for the primary endpoint, this will be handled by multiple imputation [20].

Secondary endpoints will be analyzed descriptively using regression models. Treatment effects will be calculated with two-sided 95% confidence intervals. Details will be specified in the statistical analysis plan (SAP) which will be finalized after the end of the recruitment period, but before the start of the final analysis.

Safety analyses will be performed in the safety set including all patients for whom one of the treatments was started. All safety parameters (e.g., adverse events, laboratory assessments) will be listed by the patient and displayed in summary tables. The adverse events (AEs) are displayed in summary tables by treatment as follows.

The total number of AEs; the minimum, maximum, and mean number of AEs per patient; the total number of follow-up days (number of days in the observation period); the number of AEs per FU-day; the number of patients who had at least one AE; and the number of patients who stopped treatment due to AE will be given.

The incidence of AEs defined by the preferred term (PT) according to MedDRA will be calculated as the number of patients who experienced at least one AE with the respective PT in the percentage of the total number of patients in the safety population. In the incidence tables, the PTs will be grouped by system organ class (SOC) according to MedDRA. Additionally, the incidence of AEs defined by SOC will be calculated as the number of patients who experienced at least one AE in the respective SOC as a percentage of the total number of patients in the safety population.

Each table will be produced for the following AE sets: all AEs, AEs being at least severe, serious adverse events (SAEs), SAEs leading to death, AEs possibly related to IMP, AEs possibly related to IMP being at least severe (toxicity), SAEs possibly related to IMP, and SAEs possibly related to IMP leading to death. Incidences of AEs will be calculated with 95% confidence intervals.

### Interim analyses {21b}

No interim analyses are scheduled for this trial. However, an independent data monitoring committee (DMC) periodically will receive an overview of the current safety data. Individual patients may be withdrawn from the trial if one or more of the following criteria are met:

- Withdrawal of consent on the part of the patient or legal representative
- Adverse events (including intercurrent illnesses) which preclude further treatment with the IMP or make further participation in the clinical trial inadvisable
- Premature termination considered to be medically indicated
- Pregnancy
- Significant violations of the trial protocol or lack of compliance on the part of the patient

A treatment arm or the entire clinical trial may be terminated prematurely if:

- Following a safety analysis by the DMC, the sponsor or the coordinating investigator consider that the risk-benefit ratio for trial patients has changed significantly
- Question(s) addressed in the trial can be clearly answered on the basis of the results of another trial on the same subjects
- The recruitment rate is insufficient, defined as < 3 patients recruited within the first 9 months

### Methods for additional analyses (e.g., subgroup analyses) {20b}

Not applicable, no subgroup or adjusted analysis is planned.

### Methods in analysis to handle protocol non-adherence and any statistical methods to handle missing data {20c}

Efficacy analyses will be performed primarily in the full analysis set (FAS) according to the intention-to-treat (ITT) principle. This means that the patients will be analyzed in the treatment arms to which they were randomized, irrespective of whether they refused or discontinued the treatment or whether other protocol violations occurred.

The analysis of the per-protocol (PP) population will be performed for the purpose of a sensitivity analysis. The PP population is a subset of the FAS and is defined as the group of patients who had no major protocol violations, received a predefined minimum dose of the treatment, and underwent the examinations required for the assessment of the endpoints at relevant, predefined times.

Safety analyses will be performed in the safety population. Patients in the safety population are analyzed as belonging to the treatment arm defined by treatment received. Patients are included in the respective treatment arm, if treatment was started.

### Plans to give access to the full protocol, participant level-data, and statistical code {31c}

Public access to the full protocol is granted by way of the present publication. Participant-level data may be made available upon reasonable request, and as far as permitted by data protection legislation.

## Oversight and monitoring

### Composition of the coordinating center and trial steering committee {5d}

The principal investigator and his deputy are responsible for the following:

- Design and conduct of the study and general study oversight
- Preparation of protocol and revisions
- Preparation of investigators brochure (IB)
- Publication of study reports

The Clinical Trials Unit of the Medical Center, University of Freiburg, under the supervision of the principal investigator, is responsible for the following:

- Project management, i.e., regulatory compliance, preparation of submissions to competent authorities, finance and contract management, maintenance of trial master file, coordination of study team meetings
- Data management, i.e., design, validation, and maintenance of case report forms and study database
- Pharmacovigilance, i.e., adverse event reporting to the national competent authority
- Clinical monitoring, source data verification, and quality management
- Planning and performance of statistical analyses

### Composition of the data monitoring committee, its role, and reporting structure {21a}

Additionally, an independent Data Monitoring Committee (DMC) has been established to monitor the course of the trial, to evaluate the risk threshold and the degree of distress for trial patients, and to give recommendations to the sponsor regarding discontinuation, modification, or continuation of the trial. Data evaluated by the DMC are specified in detail in a DMC charter which can be found in Additional File 2.

### Adverse event reporting and harms {22}

An adverse event (AE) is any untoward medical occurrence in a patient administered any dose of a pharmaceutical product which does not necessarily have a causal relationship with the use of the product. An AE can therefore be any unfavorable and unintended sign (including an abnormal laboratory finding), symptom, or disease temporally associated with the use of an IMP, whether or not related to the product.

In order to monitor the conditions of the patients from the time the patients receive the catheter implantation, the investigator is requested to report any untoward clinical event on the AE page of the CRF. Irrespective of any causal relationship, all AEs spontaneously reported by the patient or observed by the investigator will be documented in the case report form starting from randomization until the end of the study (month 6).

All AEs must be described by diagnosis or, if an underlying diagnosis is not known, by symptoms or medically significant laboratory or instrumental abnormalities. Symptoms, medically significant laboratory, or instrumental (e.g., electrocardiographic) abnormalities of a pre-existing disease are not considered an AE. Occurrences of new symptoms or laboratory or instrumental abnormalities, as well as worsening of pre-existing ones, are considered AEs. All AEs, no matter how intense, are to be followed up by the investigator in accordance with ICH-GCP until resolved or judged no longer clinically relevant or, in case of a chronic condition, until it is fully characterized.

For any AE, the following will be documented:

- Characterization of the event (diagnosis; if not available, symptoms)
- Onset/end date
- Severity according to the common classification: (1) mild, (2) moderate, (3) severe, (4) life-threatening/disabling, and (5) death
- Relationship to the IMP(s) (related/not related) or to study-specific procedures (such as stereotactic surgery or intrathecal lavage)
- Serious/non-serious
- Action taken with IMP(s)
- Outcome

In this clinical trial, the following adverse events will be pre-specified (e.g., as a drop-down menu) for documentation in the CRF:

- Delayed ischemic neurological deficit (DIND)
- DCI
- Hydrocephalus requiring shunt implantation
- Endovascular interventions for the treatment of cerebral vasospasm
- Complications after aneurysm securing
- Intracerebral hemorrhage
- Re-SAH
- Intracerebral infection (meningitis and/or encephalitis)
- Episodes of critically increased ICP

During each visit, it will be actively evaluated whether one of these events occurred since the last study visit.

Serious adverse events (SAEs) are subject to additional documentation and expedited reporting requirements as given by German legislation. An SAE is defined as any untoward medical occurrence that results in one or more of the following outcomes:

- Death
- Life-threatening situation (patient is at immediate risk of death), inpatient hospitalization, or prolongation of existing hospitalization (excluding those for study therapy and/or assessments, placement of an indwelling catheter, social/convenience admissions, respite care, elective or pre-planned treatment/surgery)
- Persistent or significant disability/incapacity
- Congenital anomaly/birth defect
- Other medically important conditions: conditions which, in the investigator’s opinion, may not be immediately life-threatening or result in hospitalization but may jeopardize the patient’s safety or may require intervention to prevent one of the other outcomes listed in the definition above, may also be considered serious. Examples of such conditions include allergic bronchospasm requiring treatment in an emergency room or at home, unexpected convulsions (i.e. convulsions which cannot be explained by the underlying illness) that do not result in hospitalization, development of IMP dependency or drug abuse, and suspected transmission of infectious agents by a medicinal product

In sum, established guidelines and definitions, standard operating procedures, and applicable laws and regulations will be followed in the documentation and reporting of adverse events. The results of the safety analyses described above will be reported in trial publications.

### Frequency and plans for auditing trial conduct {23}

Audits may be performed according to local quality assurance requirements in order to determine whether appropriate processes are in place and are laid down in standard operating procedures (system audit); whether such processes are carried out in a way that is reasonable, comprehensive, and in line with applicable regulation; whether the responsible personnel is appropriately qualified to take on their tasks (process audit); and whether important documents (e.g., clinical trial protocol, case report forms) comply with all relevant local and regulatory provisions (product audit). Such evaluations may take the form of internal (e.g., the Clinical Trials Unit being audited by its own quality assurance department) or external audits (e.g., the Clinical Trials Unit auditing the Department of Neurosurgery, or the central department for governance and quality of the Medical Center, University of Freiburg, auditing the Clinical Trials Unit or the Department of Neurosurgery).

### Plans for communicating important protocol amendments to relevant parties (e.g., trial participants, ethical committees) {25}

Substantial amendments to the trial protocol (e.g., changes to eligibility criteria, outcomes, analyses) will only be implemented after CA and EC approval has been obtained. Changes to the protocol that are required for patient safety may be implemented prior to CA and EC approval. In case of protocol amendments, the relevant entry in the trial registry will be updated accordingly.

### Dissemination plans {31a}

Upon trial completion, the results of this trial will be submitted for publication in a scientific journal, presented at academic conferences, and posted in a publicly accessible database of clinical trial results irrespective of the results of the trial. No contractual restrictions exist regarding the publication of trial results. In line with ICMJE recommendations on authorship, the authors of the main publication shall be all those who have made significant intellectual contributions to the design and conduct of the study, or to acquiring, interpreting, and analyzing the study data. It is not intended to engage professional writers for publications of this trial.

## Discussion

DCI is a major cause of death and poor neurological outcome in patients with aSAH. The therapeutic armamentarium currently available to address this problem is narrow and insufficient. Thus, there is a significant need to develop new treatment approaches for these patients.

Direct intrathecal therapies with fibrinolytic (e.g., urokinase) and spasmolytic agents (e.g., nimodipine) have appeared promising in clinical trials. However, in patients with aneurysms secured by endovascular intervention (coiling) which is the dominant technique of aneurysm securing today, no safe method of access to the very target of this therapy exists.

We introduce STX-VCS to establish a cisternal treatment access irrespective of the method of aneurysm securing and propose a stepwise protocol for cisternal drug delivery. The pathophysiologic rationale of our approach is to remove the presumed trigger for CVS through fibrinolysis of subarachnoid blood clots and to target CVS directly in case it occurs. Furthermore, as ventricular blood presents an add-on risk for vasospasm [21], it appears plausible to involve the ventricles in the lavage circuit and hence to establish a ventriculo-cisternal lavage. We propose this RCT to assess the clinical safety and efficacy of this approach.

Additionally, the majority of DCI cases occur in patients of WFNS grade 5 (68% of DCI cases in our institutional series). By including WFNS 5 patients, the present trial is designed to be offered to (nearly) all patients at risk for DCI, i.e., WFNS 3–5. This approach differs from most other recent and ongoing RCTs on CVS and DCI which typically exclude WFNS 5 patients.

## Trial status

The study is recruiting patients since June 12, 2019. We expect recruitment to be completed by late 2022.

## Supplementary Information

**Additional file 1:** Visit schedule and assessments.

**Additional file 2:** DMC charter.

## Abbreviations

AE: Adverse event
CA: Competent authority
CSF: Cerebrospinal fluid
(c)CT: (Cranial) computed tomography
CVS: Cerebral vasospasm
DCI: Delayed cerebral infarction
DIND: Delayed ischemic neurological deficit
DMC: Data monitoring committee
EC: Ethics committee
EEG: Electroencephalography
EVD: External ventricular drain
FAS: Full analysis set
GCS: Glasgow Coma Scale
ICH-GCP: International Council for Harmonization – Good Clinical Practice guideline
ICP: Intracranial pressure
IMP: Investigational medicinal product
IT: Investigational treatment
ITT: Intention-to-treat
I.U.: International units
MedDRA: Medical Dictionary for Regulatory Activities
MRI: Magnetic resonance imaging
mRS: Modified Rankin Scale
NIHSS: National Institutes of Health Stroke Scale
PP: Per-protocol
PT: Preferred term (MedDRA term)
RCT: Randomized controlled trial
rtPA: Recombinant tissue plasminogen activator
SAE: Serious adverse event
(a)SAH: (Aneurysmal) subarachnoid hemorrhage
SOC: System organ class (MedDRA term)
SOFA: Sequential organ failure assessment
STX-VCS: Stereotactic catheter ventriculocisternostomy
WFNS: World Federation of Neurosurgical Societies

## References

[1] Macdonald RL, Schweizer TA (2017). Spontaneous subarachnoid haemorrhage. Lancet.

[2] Vergouwen MDI, Etminan N, Ilodigwe D, Macdonald RL (2011). Lower incidence of cerebral infarction correlates with improved functional outcome after aneurysmal subarachnoid hemorrhage. J Cereb Blood Flow Metab Off J Int Soc Cereb Blood Flow Metab.

[3] Jabbarli R, Reinhard M, Roelz R, Shah M, Niesen W-D, Kaier K, Taschner C, Weyerbrock A, Velthoven VV (2015). Early identification of individuals at high risk for cerebral infarction after aneurysmal subarachnoid hemorrhage: the BEHAVIOR score. J Cereb Blood Flow Metab Off J Int Soc Cereb Blood Flow Metab..

[4] van Gijn J, Kerr RS, Rinkel GJ (2007). Subarachnoid haemorrhage. Lancet.

[5] Velat GJ, Kimball MM, Mocco JD, Hoh BL (2011). Vasospasm after aneurysmal subarachnoid hemorrhage: review of randomized controlled trials and meta-analyses in the literature. World Neurosurg.

[6] Macdonald RL, Pluta RM, Zhang JH (2007). Cerebral vasospasm after subarachnoid hemorrhage: the emerging revolution. Nat Clin Pract Neurol.

[7] Findlay JM, Nisar J, Darsaut T. Cerebral vasospasm: a review. Can J Neurol Sci J Can Sci Neurol. 2016;43:15–32. 10.1017/cjn.2015.288 26332908

[8] Dorhout Mees SM, Rinkel GJE, Feigin VL, Algra A, van den Bergh WM, Vermeulen M, et al. Calcium antagonists for aneurysmal subarachnoid haemorrhage. Cochrane Database Syst Rev. 2007;3:CD000277. 10.1002/14651858.CD000277.pub3 PMC7044719 17636626

[9] Pickard JD, Murray GD, Illingworth R, Shaw MD, Teasdale GM, Foy PM, Humphrey PR, Lang DA, Nelson R, Richards P (1989). Effect of oral nimodipine on cerebral infarction and outcome after subarachnoid haemorrhage: British aneurysm nimodipine trial. BMJ..

[10] Gathier CS, van den Bergh WM, van der Jagt M, Verweij BH, Dankbaar JW, Müller MC, Oldenbeuving AW, Rinkel GJE, Slooter AJC, Algra A, Kesecioglu J, van der Schaaf IC, Dammers R, Dippel DWJ, Dirven CMF, van Kooten F, van der Lugt A, Coert BA, Horn J, Vandertop WP, Beute GN, van der Pol B, Roks G, van Rooij WJJ, Sluzewski M, for the HIMALAIA Study Group* (2018). Induced hypertension for delayed cerebral ischemia after aneurysmal subarachnoid hemorrhage: a randomized clinical trial. Stroke..

[11] Kimball MM, Velat GJ, Hoh BL, Participants in the International Multi-Disciplinary Consensus Conference on the Critical Care Management of Subarachnoid Hemorrhage (2011). Critical care guidelines on the endovascular management of cerebral vasospasm. Neurocrit Care.

[12] Zaidi HA, Montoure A, Elhadi A, Nakaji P, McDougall CG, Albuquerque FC (2015). Long-term functional outcomes and predictors of shunt-dependent hydrocephalus after treatment of ruptured intracranial aneurysms in the BRAT Trial: revisiting the clip vs coil debate. Neurosurg Oxford Acad.

[13] O’Kelly CJ, Kulkarni AV, Austin PC, Urbach D, Wallace MC (2009). Shunt-dependent hydrocephalus after aneurysmal subarachnoid hemorrhage: incidence, predictors, and revision rates. Clinical article. J Neurosurg.

[14] Zhang YP, Shields LBE, Yao TL, Dashti SR, Shields CB (2013). Intrathecal treatment of cerebral vasospasm. J Stroke Cerebrovasc Dis Off J Natl Stroke Assoc.

[15] Etminan N, Beseoglu K, Eicker SO, Turowski B, Steiger H-J, Hänggi D (2013). Prospective, randomized, open-label phase II trial on concomitant intraventricular fibrinolysis and low-frequency rotation after severe subarachnoid hemorrhage. Stroke..

[16] Hänggi D, Eicker S, Beseoglu K, Behr J, Turowski B, Steiger H-J (2009). A multimodal concept in patients after severe aneurysmal subarachnoid hemorrhage: results of a controlled single centre prospective randomized multimodal phase I/II trial on cerebral vasospasm. Cent Eur Neurosurg.

[17] Roelz R, Coenen VA, Scheiwe C, Niesen W-D, Egger K, Csok I, Kraeutle R, Jabbarli R, Urbach H, Reinacher PC (2017). Stereotactic catheter ventriculocisternostomy for clearance of subarachnoid hemorrhage: a matched cohort study. Stroke..

[18] Steiner T, Juvela S, Unterberg A, Jung C, Forsting M, Rinkel G, European Stroke Organization (2013). European Stroke Organization guidelines for the management of intracranial aneurysms and subarachnoid haemorrhage. Cerebrovasc Dis Basel Switz.

[19] Roelz R, Scheiwe C, Urbach H, Coenen VA, Reinacher P (2018). Stereotactic catheter ventriculocisternostomy for clearance of subarachnoid hemorrhage in patients with coiled aneurysms. Oper Neurosurg Oxford Academic.

[20] Rubin DB (1987). Multiple imputation for nonresponse in surveys.

[21] Claassen J, Bernardini GL, Kreiter K, Bates J, Du YE, Copeland D (2001). Effect of cisternal and ventricular blood on risk of delayed cerebral ischemia after subarachnoid hemorrhage: stroke. Am Heart Assoc.

