# Supplementary material for: Stereotactic cisternal lavage in patients with aneurysmal subarachnoid hemorrhage with urokinase and nimodipine for the prevention of secondary brain injury (SPLASH): study protocol for a randomized controlled trial
Source: Trials. 2021 Apr 15;22:285. doi: 10.1186/s13063-021-05208-6 (PMC8048077; doi:10.1186/s13063-021-05208-6)
Supplement: Supplementary file 1 — Additional file 1: Visit schedule and assessments. [file 13063_2021_5208_MOESM1_ESM.docx]

Visit schedule and assessments

| Assessment | Comment / explanation | Timeline | | | | | | |
| --- | --- | --- | --- | --- | --- | --- | --- | --- |
|  |  | aSAH event | Screening | Rando-mization | aSAH treatment | post aSAH treatment /  Follow-up | | |
|  |  | Day 0 | Day 2±2 ≤96h after aSAH | | From randomization until day 21 | Day 32±10 | Dis-charge | End of Study / Month 6  (± 2 weeks) |
| Informed Consent |  |  | X |  |  |  |  |  |
| Inclusion / Exclusion Criteria |  |  | X |  |  |  |  |  |
| Randomization |  |  |  | X |  |  |  |  |
| Demographics |  |  | X |  |  |  |  |  |
| Medical History |  |  | X |  |  |  |  |  |
| Pregnancy test |  |  | X |  |  |  |  |  |
| Concomitant medication |  |  | X |  | daily | X |  | X |
| Neurological examination  (GCS, NIHSS) | GCS and NIHSS are well-established scores in the clinical assessment of (neuro-) intensive care patients. |  | X |  | daily | X |  | X |
| Assessment of initial routine cCT | The following parameters will be documented:  Modified Fisher grade, Hijdra Score, presence of intracerebral hemorrhage (yes/no), volume estimation of intracerebral hemorrhage if present, location of intracerebral hemorrhage if present, signs of cerebral infarction (yes/no), size (mm) and location of ruptured aneurysm, presence of multiple aneurysms (yes/no). |  | X |  |  |  |  |  |
| Planning imaging scan  (CT and/or MRI) | group 2 only |  |  |  | once: a.s.a.p. after randomization |  |  |  |
| Stereotactic neurosurgery /  STX-VCS catheter implantation | group 2 only |  |  |  | once: a.s.a.p. after randomization |  |  |  |
| Postoperative cCT | Every patient, regardless of trial arm, receives a clinical routine cCT scan between 24 and 48h after aneurysm treatment (coiling/clipping) to control for appropriate aneurysm securing **(standard post aneurysm treatment cCT)**. In group 2, an additional **postoperative cCT** to control for correct STX-VCS catheter placement is necessary after stereotactic neurosurgery. If performed between 24 and 48h after aneurysm treatment, the postoperative cCT in group 2 can be used as standard post aneurysm treatment CT. Otherwise, a separate post-treatment CT scan is necessary in group 2. As the post-aneurysm treatment CT is a routine scan and must be performed primarily as clinically indicated (within 48 hours after aneurysm securing), this CT may also be performed before randomization, i.e. within the screening phase. Most notably, this will be the case when randomization occurs later than 48 hours after aneurysm securing. |  |  |  | once: a.s.a.p. after STX-VCS catheter implantation |  |  |  |
| Standard post aneurysm treatment cCT |  |  |  |  | once: 24-48h after aneurysm treatment (coiling / clipping) |  |  |  |
| IMP administration | group 2 only |  |  |  | daily / continuous |  |  |  |
| Transcranial Doppler Ultra-Sonography | The following parameters will be documented: Mean flow velocities (cm/s) of left and right intracranial internal carotid arteries (ICA), middle cerebral arteries (MCA), anterior cerebral arteries (ACA), posterior cerebral arteries (PCA) and the basilar artery (BA); delta mean flow velocities (ΔMFV, %) between any two consecutive days; Lindegaard-Index in case of significant ΔMFV |  |  |  | daily |  |  |  |
| Blood samples | Neuron-specific enolase, S100 calcium-binding protein,  Interleukin 6, C-reactive protein (CRP) |  |  |  | day 2±2, 7, 14 |  |  |  |
| Parameters of Intensive Care Medicine (SOFA) | SOFA is an established clinical score to predict mortality of intensive care patients based on laboratory and clinical data, e.g. parameters of renal, hepatic and respiratory function. |  |  |  | daily |  |  |  |
| EEG monitoring | In selected patients only (exploratory endpoint). Pathological electroencephalographic patterns such as periodic lateralized discharges, lateralized rhythmic delta activity, cortical spreading depression will be evaluated. |  |  |  | at selected points in time during intensive care period |  |  |  |
| DCI-rating CT-scan | CT scan for DCI rating by an interdisciplinary board will be performed for patients in both groups. DCI is rated according to the Vergouwen criteria. |  |  |  |  | X |  |  |
| mRS | The modified Rankin Scale (mRS) is a commonly used scale for measuring the degree of disability or dependence in the daily activities of people who have suffered a stroke or other causes of neurological disability. It has become the most widely used clinical outcome measure for stroke in clinical trials. The scale runs from zero (perfect health without symptoms) to six (death). mRS scoring will be performed by a qualified and independent rater who is otherwise not involved in the study. |  |  |  |  | X |  | X |
| Endocrinological assessments | The function and deficits of the pituitary hormonal axes will be assessed by specific laboratory testing using baseline parameters as well as a combined pituitary stimulation test with releasing hormones.  Specifically, the following functions will be assessed: Adrenocorticotropic function, thyrotropic function, somatotropic function, mammotropic function, gonadotropic function. |  |  |  |  |  |  | X |
| Date of discharge |  |  |  |  |  |  | X |  |
| Neuropsychological  assessments | Established and validated instruments will be used to assess cognitive performance (Montreal Cognitive Assessment), fatigue, anxiety, depressive symptoms and symptoms of post-traumatic stress disorder (Frontal Systems Behavior Scale, Multidimensional Assessment of Fatigue, Hospital Anxiety and Depression Scale, Impact of Event Scale - R), quality of life (Short Form-36 Health Survey), and return-to-work-parameters (employment and weekly working hours before and after aSAH, time between aSAH and return to work) |  |  |  |  |  |  | X |
| Neuroradiological outcome (MRI) | The MRI will be analysed descriptively with regard to neuroradiological outcome. |  |  |  |  |  |  | X |
| Shunt-dependent hydrocephalus (y/n) |  |  |  |  |  |  |  | X |
| Adverse Events |  |  |  |  | daily | X | X | X |
