# Supplementary material for: Stereotactic cisternal lavage in patients with aneurysmal subarachnoid hemorrhage with urokinase and nimodipine for the prevention of secondary brain injury (SPLASH): study protocol for a randomized controlled trial
Source: Trials. 2021 Apr 15;22:285. doi: 10.1186/s13063-021-05208-6 (PMC8048077; doi:10.1186/s13063-021-05208-6)
Supplement: Supplementary file 2 — Additional file 2: DMC charter. [file 13063_2021_5208_MOESM2_ESM.pdf]

Stereotactic Cisternal Lavage in Patients with Aneurysmal Subarachnoid Hemorrhage with Urokinase and Nimodipine for the Prevention of Secondary Brain Injury. A Randomized Controlled Trial.

-

SPLASH

**EudraCT number:**	**2017-000868-15**
**Protocol code:**	**P001151**
**Sponsor:**	**Medical Center - University of Freiburg**<br>represented by the Chief Medical Officer (CMO)<br>(Leitender Ärztlicher Direktor)<br>Breisacher Str. 153, 79110 Freiburg<br>*all tasks delegated to **Principal Investigator***
**Principal Investigator:**	**Dr. Roland Roelz**
Institution:	Department of Neurosurgery
Address:	Breisacher Str. 64, 79106 Freiburg
Phone:	+49 761 270-50940
Fax:	+49 761 270-50100
Email:	roland.roelz@uniklinik-freiburg.de

Data Monitoring Committee (DMC)

– Charter –

Version-No.: 01.3

Date: 05 May 2020

Table of Contents

1. Introduction 3
2. Composition of the DMC 3
2.1. DMC members 3
2.2. DMC chairperson 3
3. Responsibilities of the DMC 4
4. Regular meetings, documentation and reports 4
4.1. Meetings 4
4.2. Documentation and reporting 5
4.3. Unscheduled meetings 5
5. Data provided to the DMC 5
6. Conflicts of interest 6
7. Confidentiality 6
8. Signatures 7
9. Appendix 1: DMC Recommendation 8

1. Introduction

This charter is set up for the Data Monitoring Committee (DMC) of the clinical trial "Stereotactic Cisternal Lavage in Patients with Aneurysmal Subarachnoid Hemorrhage with Urokinase and Nimodipine for the Prevention of Secondary Brain Injury. A Randomized Controlled Trial". It describes composition and responsibilities of the DMC and includes details on regular meetings, reporting requirements and communication lines. This document further provides details on monitoring of Serious Adverse Events (SAE) and subsequent actions, as well as details on interim analyses.

2. Composition of the DMC

The DMC consists of 3 voting members: a neurologist, a neurosurgeon and a biostatistician.

2.1. DMC members

The voting members for this clinical trial are:

1. Neurologist

Name [redacted]
Address
Institution
Phone
Fax
E-mail

2. Neurosurgeon

Name [redacted]
Address
Institution
Phone
Fax
E-mail

3. Biostatistician

Name [redacted]
Address
Institution
Phone
Fax
E-mail

2.2. DMC chairperson

The DMC chairperson will be named at the constitutional meeting.

3. Responsibilities of the DMC

The DMC gives recommendations to the sponsor and the principal investigator regarding patient safety in this clinical trial.

For this purpose the DMC will review the results of safety analyses and regular safety reports at specified intervals. The DMC will assess the benefit-risk-profile of the study treatment in the trial population. As an additional focus of its task, the DMC will pay attention to the degree of distress and the risk threshold for trial patients based on a comparison of AE / SAE rates in both trial arms. Furthermore, the DMC may evaluate adherence to the trial protocol.

The DMC will discuss the trial conduct with respect to the following aspects:

- Increased incidence of SAEs / unacceptable toxicities
- Comparison of AE / SAE rates in both trial arms
- Unexpected adverse reactions that lead to a consecutive change in the benefit-risk-assessment

The DMC will make recommendations in accordance with the criteria established by the DMC or the sponsor with regard to conduct or design of the clinical trial, including design modifications and early termination. A DMC recommendation form template will be provided in Appendix 1.

If possible, the decision for an early termination of the clinical trial will be made in consultation with the sponsor, the principal investigator and his deputy and the DMC. The responsibility for the final decision lies with the sponsor.

4. Regular meetings, documentation and reports

4.1. Meetings

The DMC will schedule periodic meetings while the trial is ongoing. During the conduct of the trial, it is planned to organize one meeting at every time that six patients have finished their treatment phase and entered the follow-up phase, starting from First Patient In. That is, the first periodic meeting will take place after day 32 of patient 06, the next one after day 32 of patient 12, and so on.

All meetings will be organised by the Project Manager (PM) of the Clincal Trials Unit of the Medical Center, University of Freiburg, as telephone conferences or face-to-face meetings.

Prior to the first meeting of the DMC there will be a constitutional meeting to allow members to discuss and approve organisational issues.

Each meeting consists of two parts:

- An open session for data review and discussion of the trial status with the LKP
- A closed session that is restricted to the 3 DMC members for further discussion and independent decision regarding their recommendations.

The DMC will maintain confidentiality of all information received and discussed in meetings.

4.2. Documentation and reporting

Minutes of the open sessions will be prepared and circulated by the PM. The final minutes will be filed to the TMF.

Meeting minutes of the closed session will be prepared by one of the DMC members. These meeting minutes will be retained separately from open session minutes by the DMC chair person until the end of the trial.

At each meeting, the DMC makes a recommendation regarding further trial continuation. This may include any aspect in conduct or design of the clinical trial, design modification and early termination, or aspects regarding the degree of distress and the risk threshold of trial patients. The chairperson will communicate recommendations to the sponsor, the principal investigator and his deputy and to the responsible PM. The recommendation will be filed to the TMF.

4.3. Unscheduled meetings

In the event of unforeseen circumstances, such as unexpected serious adverse drug reactions, the sponsor or the DMC members may request an unscheduled meeting of the DMC.

5. Data provided to the DMC

The DMC will receive the following data for evaluation prior to each meeting:

- SAE listings grouped by treatment arm and information on SUSARs

  These data will be provided as line listings and summary tabulation according to DSUR format by the pharmacovigilance staff of the CTU.

- Number of:
  - Recruited patients
  - Drop-outs / early termination
- Protocol deviations
- mRS and neurological examinations at day 32 and 6 months follow-up visits (as far as available)
- All adverse events (including non-serious)

  These data will be provided by the data management staff of the CTU.

Additionally, SARs, SUSARs and SAE related to stereotactic surgery (i.e. serious surgical complications) will be reported to the DMC as timely as possible upon awareness.

If required, the following data can be made available upon request of the DMC:

- Discharge letters and letters of further outpatient and inpatient visits at the trial site
- All available cranial imaging (e.g. CT and MRI) and corresponding radiological reports
- All available operative notes / notes of endovascular interventions

  Discharge letters, letters of outpatient and inpatient visits and notes on surgery and interventios can be provided as printed copy by the study assistant / study nurse. Personally identifying information (names, birth dates) will be blackened. Imaging data and reports can be provided in anonymized form by the neuroradiologist as a digital copy (CD).

6. Conflicts of interest

By signing this document, the DMC members declare that they do not have any conflicts of interest, or to disclose any conflicts of interest, whether they are of financial, intellectual, professional or scientific nature.

Members must disclose any present, developing or any foreseen upcoming conflict(s) of interest; the respective document(s) will be appended to this charter.

7. Confidentiality

The DMC will maintain confidentiality of all information received. This includes documents such as meeting minutes prepared by the DMC, reports, statistical calculations etc.

Each member of the DMC has to retain the received documents and has to keep them confidential until the final study report has been published.

DMC members sign a confidentiality disclosure agreement (CDA).

8. Signatures

By signing this document the following persons agree to this DMC charter:

**Sponsor** *(delegated to Principal Investigator)*		
Dr. Roland Roelz	Date	Signature

**Trial biostatistician**		
Dr. Carolin Jenkner	Date	Signature

**DMC Neurologist**		
[redacted]	Date	Signature

**DMC Neurosurgeon**		
[redacted]	Date	Signature

**DMC Biostatistician**		
[redacted]	Date	Signature

9. Appendix 1: DMC Recommendation

Data Monitoring Committee Recommendation

EudraCT number: **2017-000868-15**

Protocol code: **P001151**

From: Data Monitoring Committee Chair: <insert chairperson>

To: Sponsor *(delegated to Principal Investigator):* Dr. Roland Roelz

CC: Deputy, Project manager

On dd.mm.yyyy <insert date of meeting> the Data Monitoring Committee has reviewed the data summary dated dd.mm.yyyy <insert date of DMC data set>.

Based on our review, we recommend:

☐	1. The study may continue without modifications.
☐	2. The study may continue with modifications (see report).
☐	3. The study should be stopped (see report).
☐	4. Issues with regard to the risk threshold and/or degree of distress of the patients require correction (see report).
☐	5. More data are required for review (see report).

Report:

Signature Chairperson

Date (dd.mm.yyyy)
